# An in vitro comparison of standard cleaning to a continuous passive disinfection cap for the decontamination of needle-free connectors

**DOI:** 10.1186/s13756-018-0342-0

**Published:** 2018-04-05

**Authors:** Anna L. Casey, Tarja J. Karpanen, Peter Nightingale, Tom S. J. Elliott

**Affiliations:** University Hospitals Birmingham NHS Foundation Trust, Birmingham, B15 2TH UK

**Keywords:** Disinfection cap, Wipe, Needle-free connectors, Isopropyl alcohol, Chlorhexidine

## Abstract

**Background:**

The optimal decontamination method for needle-free connectors is still unresolved. The objective of this study was to determine if a continuous passive disinfection cap is as effective as standard cleaning for the microbial decontamination of injection ports of two types of needle-free connectors.

**Methods:**

The injection ports of needle-free connectors were inoculated with *Staphylococcus aureus* and allowed to dry. Disinfection caps containing 70% (*v*/*v*) isopropyl alcohol (IPA) were attached to the connectors for one, three or 7 days and were compared with needle-free connectors cleaned with 2% (*w*/*v*) chlorhexidine gluconate (CHG) in 70% (*v/v*) IPA. The number of *S. aureus* remaining on the injection ports was evaluated. Median log_10_ reductions and 95% confidence interval (CI) were calculated and data analyzed using the Mann-Whitney test.

**Results:**

The application of the disinfection cap resulted in a significantly higher reduction in *S. aureus* than the 2% (*w*/*v*) CHG in 70% (*v*/*v*) IPA wipe, achieving a > 5 Log_10_ reduction in CFU at each time point.

**Conclusions:**

The disinfection caps resulted in a significantly higher reduction in *S.aureus* on the injection ports when compared to the use of a 2% (*w/v*) CHG in 70% (*v/v*) IPA wipe. This offers an explanation for the lower rates of central-line associated bloodstream infection (CLABSI) associated with the use of disinfection caps reported in clinical studies.

## Background

There have been varying reports on the rates of bloodstream infection (BSI) associated with needle-free connectors including an increase in incidence following a change from split-septum connectors to mechanical connectors [1]. The Centers for Disease Control and prevention (CDC) has subsequently recommended that when needleless systems are used, a split-septum valve may be preferred over some mechanical valves [2]. Furthermore, The Society for Healthcare Epidemiology of America (SHEA) and the Infectious Diseases Society of America (IDSA) advised that positive-pressure needleless connectors with mechanical valves should not be used before a thorough assessment of risks, benefits, and education regarding proper use [3]. The FDA requested that manufacturers of positive-displacement devices should conduct post-market surveillance to demonstrate that their devices were not associated with an increased risk of BSI compared to other types of device. A SHEA/IDSA practice update subsequently stated that the optimal needle-free connector design for the prevention of infection was still unresolved and an assessment of risks, benefits and education was again recommended [4].

Many factors have been attributed to the level of infection risk associated with needle-free connectors and includes the efficacy of disinfection of the injection ports [5]. It has also been suggested that surface disinfection of needle-free connectors is not intuitive which may lead to non-compliance [5].

Caps which attach to injection ports of needle-free connectors incorporating disinfectants have been developed. Menyhay and Maki described such a device containing 2% chlorhexidine gluconate (CHG) in 70% isopropyl alcohol (IPA) in 2006 [6]. The caps act as passive disinfection devices which are designed to ensure that needle-free connectors are always clean.

Several clinical studies have evaluated the use of these passive disinfection devices, all of which demonstrate benefits including significant reductions in the rates of hub microbial colonisation [7], and central-line associated bloodstream infections (CLABSI) [8–12].

Whilst these studies represent the clinical scenario whereby adherence to decontaminating the needle-free connector may not always be optimal, they do not investigate the efficacy of a defined cleaning method compared to passive disinfection caps under optimal, controlled conditions.

The aim of the study was to determine under controlled laboratory conditions whether a commercially available continuous passive disinfection cap which contains 70% (*v/v*) IPA was as effective for microbial decontamination of two different needle-free connectors when compared to defined standard cleaning with a 2% (*w*/*v*) CHG in 70% (*v*/*v*) IPA wipe.

## Methods

### Needle-free connectors and cleaning devices

The needle-free connectors used in this study were a neutral displacement connector - MicroClave™ (ICU Medical) and a positive-displacement connector - CareSite™ (BBraun). Curos® caps containing 70% (*v/v*) IPA (3 M Healthcare) were compared to 2% (*w*/*v*) CHG in 70% (*v/v*) IPA wipes (Sani-cloth CHG 2%, PDI) for decontamination of the needle-free connectors.

### Contamination of needle-free connectors

An overnight culture of *Staphylococcus aureus* National Collection of Type Cultures (NCTC) 6538 on tryptic soy agar (Oxoid) was used to prepare a 1 × 10^8^ CFU/mL suspension in tryptone sodium chloride (1 g/L tryptone [Oxoid], 8.5 g/L NaCl [Sigma-Aldrich] in distilled water) containing 3 g/L bovine albumin faction V [VWR International] and 3 ml/L defibrinated sheep blood [TCS Biosciences] in accordance with BS EN 16615:2015 [13].

Following one activation of each connector, the external injection port of each sterile needle-free connector were inoculated with a 50 μL suspension containing at least 5 × 10^6^ CFU of *S. aureus* and allowed to air dry for 4 h at 20 °C. Whilst the high inoculum of *S.aureus* used in this study would not be expected in the clinical scenario, it permitted the identification of any differences present between the two decontamination methods, was also representative of European standard antiseptic test conditions [13], and simulated a worst-case scenario in the clinical situation.

### Evaluation of the variability in wiping technique by different operators

Needle-free connectors were cleaned for 15 s (through 180° 15 times) with a 2% (*w*/*v*) CHG in 70% (*v*/*v*) IPA wipe and allowed to dry for 30 s (this method was completed independently by two different experienced operators). A total of 54 of each type of needle-free connnector were studied (27 of each needle-free connector by each operator).

### Evaluation of the prolonged effect of the decontamination procedures

Disinfection caps were attached to the needlefree-connectors for 1, 3 or 7 days and were compared with needle-free connectors cleaned with a 2% (*w*/*v*) CHG in 70% (*v/v*) IPA wipe. All the needle-free connectors were subsequently left at 20 °C in air for 1, 3 or 7 days. A total of 54 of each needle-free connector were studied per time point following each decontamination procedure. An identical number of control needle-free connectors which were contaminated as above and which were not decontaminated were also similarly studied and acted as positive controls for each sampling point.

### Evaluation of the effect of a pre- and post-device activation wipe

Following contamination with *S. aureus*, 54 of each type of needle-free connector were cleaned as above for 15 s with a 2% (*w*/*v*) CHG in 70% (*v/v*) IPA wipe and allowed to dry for 30 s. These were then incubated for 7 days at 20 °C and then cleaned again with a 2% (*w/v*) CHG in 70% (*v/v*) IPA wipe prior to microbiological sampling.

### Microbiological sampling of needle-free connectors

Needle-free connectors were immersed into bijous containing 1 mL of neutralizing solution consisting of 30 g/L Tween 80, 30 g/L saponin, 3 g/L lecithin, 1 g/L L-histidine, 5 g/L sodium thiosulphate in tryptone sodium chloride (all VWR International). Nullification of antimicrobial activity and non-microbial toxicity was verified prior to commencement of the study (unpublished data). The bijous were then sonicated for 10 min at 50 Hz. The entire volume of neutralizing solution was inoculated (in addition to dilutions from positive control connectors) onto chromogenic *S. aureus* plates (ChromID *S. aureus* [Biomerieux]) in duplicate.

### Sample size calculation and statistical analysis

The aim of the study sample size was to demonstrate that each decontamination method achieved a 5 log_10_ reduction in the number of *S. aureus* (or 99.999% reduction). Based on preliminary work, it was concluded that 54 of each type of needle-free connector in each scenario should give at least a 90% chance of achieving a 5 log_10_ reduction in CFU. Median log_10_ reductions and 95% confidence interval (CI) were calculated and data analyzed using the Mann-Whitney test. The level of significance was < 0.05.

## Results

### CFU counts on positive control needle-free connectors

The minimum CFU count on the controls (the needle-free connectors which were not decontaminated after inoculation with *S. aureus*) during the study was 5.17 log_10_ CFU for MicroClave™ and 5.49 log_10_ CFU for CareSite™ therefore total kill (TK) always represented a ≥ 5.17 or ≥ 5.49 log_10_ CFU reduction, respectively.

### Evaluation of the variability in wiping technique by different operators

There was no significant difference between the two operators in terms of log_10_ CFU reduction of *S. aureus* following 15 s decontamination with a 2% (*w*/*v*) CHG in 70% (*v/v*) IPA wipe and drying for 30 s for both the MicroClave™ (4.69, 95% CI = 3.56–5.29 vs 4.61, 95% CI = 3.99–5.21, *P* = 0.73) and CareSite™ (5.10, 95% CI = 4.11-TK vs 5.10, 95% CI = 3.04-TK, *P* = 0.32). Furthermore, there was no difference in the overall log_10_ CFU reduction between the two different types of needle-free connectors (*P* = 0.18 for MicroClave™ and *P* = 0.70 for CareSite™).

### Evaluation of the decontamination procedures on *S. aureus* counts.

The median and 95% CI log_10_ CFU reduction in *S. aureus* after decontamination for 15 s with a 2% (*w*/*v*) CHG in 70% (*v/v*) IPA wipe followed by incubation at room temperature for 1, 3 or 7 days or after application of the disinfection cap for 1, 3 or 7 days is shown in Table 1. The application of the disinfection cap resulted in a significantly higher log_10_ CFU reduction of the *S. aureus* than the 2% (*w/v*) CHG in 70% (*v/v*) IPA wipe, achieving a > 5 log_10_ reduction in CFU at each time point. Furthermore, there was no difference in the log_10_ CFU reduction of *S. aureus* between the two different types of needle-free connectors with any decontamination regime at any time-point.

**Table 1:** Median (95%CI) log_10_ reductions of CFU of *Staphylococcus aureus* on two types of needle-free connectors injection ports after 1, 3 and 7 days following two decontamination methods

Day	Decontamination method	Connector studied: MicroClave®	Comparison of wipe vs disinfection cap (*P* value)	Connector studied: CareSite®	Comparison of wipe vs disinfection cap (*P* value)	Comparison of MicroClave® vs CareSite® (*P* value)
1	2% (*w/v*) CHG in 70% (*v/v*) IPA wipe	> 6.45^a^ (4.97-TK)	< 0.0001^b^	TK (4.29-TK)	< 0.0001^b^	0.49
	Disinfection cap	TK (TK-TK)		TK (TK-TK)		0.75
3	2% (*w/v*) CHG in 70% (*v/v*) IPA wipe	4.66 (4.34–4.95)	< 0.0001^b^	4.77 (4.39–5.68)	< 0.0001^b^	0.98
	Disinfection cap	TK (TK-TK)		TK (TK-TK)		0.057
7	2% (*w/v*) CHG in 70% (*v/v*) IPA wipe	TK (TK-TK)	< 0.0001^b^	TK (5.20-TK)	< 0.0001^b^	0.15
	Disinfection cap	TK (TK-TK)		TK (TK-TK)		1.00

^a^the median was half-way between the values of 6.45 and total kill (TK)

^b^The reductions were greater for the disinfection cap

### Evaluation of the effect of a pre- and post-device activation wipe

Decontamination of both types of needle-free device with a 2% (*w*/*v*) CHG in 70% (*v/v*) IPA wipe both following inoculation with *S. aureus* and following each subsequent incubation period resulted in a higher log_10_ CFU reduction as compared to only cleaning following contamination for MicroClave™ only (*P* = 0.009). However, in line with the above findings, the disinfection cap still resulted in a significantly higher log_10_ CFU reduction (Table 1) as compared to the two decontaminations with 2% (*w*/*v*) CHG in 70% IPA (*v/v*) wipes for both needle-free connectors (MicroClave™ *P* = 0.041, CareSite™ *P* < 0.0001, median [95% CI] = TK [TK-TK] for both types of connector).

## Discussion

This study demonstrated that under controlled laboratory conditions a disinfection cap containing 70% (*v/v*) IPA was more effective at reducing microbial contamination of contaminated injection ports of needle-free connectors when compared to cleaning with 2% (*w*/*v*) CHG in 70% (*v/v*) IPA wipes even for 15 s. Indeed, the study demonstrated that the caps were associated with a significantly higher log_10_ CFU reduction than a 2% (*w/v*) CHG in 70% (*v/v*) IPA wipe at 1, 3 and 7 days and a two-clean regime used at 7 days. This was the case for both types of needle-free connectors tested during this study, demonstrating the efficacy across more than one specific device. Indeed, no differences in log_10_ CFU reductions between these devices were observed. The reasons for this difference in efficacy of the cap versus wipe is unresolved but may reflect the continuous antimicrobial activity of the decontamination offered by the caps rather than the relatively short time following the wipes. Another confounding factor is compliance to decontamination of needle-free connectors in clinical practice. Adherence to recommended decontamination procedures by healthcare workers prior to access of needle-free connectors has been reported to be as low as 10% [14], whereas with the use of caps compliance has been high [8–10]. Indeed, the enhanced efficacy of the caps has also been reflected in decreased rates of CLABSI with increasing cap compliance [11, 15].

It is therefore conceivable that not only the improved antimicrobial activity of the caps versus wipes together with high levels of compliance with disinfection caps may both in part account for the lower rates of CLABSI associated with their use reported in previous clinical studies.

It could also be concluded that given the significant log_10_ CFU reductions observed with the 2% (*w*/*v*) CHG in 70% (*v/v*) IPA wipe in this study, there is no requirement for the additional efficacy of the disinfection cap. However, if compliance with the use of wipes is low, the disinfection caps could prove a useful tool. Furthermore, since there is concern surrounding potential chlorhexidine- and cross-resistance to antibiotics [15, 16], the use of a decontamination regime in the absence of chlorhexidine (such as the disinfection cap used in this study) may be appealing.

A potential limitation of this current study is that the selected single decontamination of the injection ports with a wipe may not be representative of the frequency with which this would occur in the clinical scenario. Similarly, if the disinfection caps were employed in the inpatient clinical scenario they would be accessed and replaced more frequently. This would be the case in clinical areas where IV devices are frequently accessed such as in critical care. However, the selected decontamination regimen used in this current study is representative of the outpatient scenario where central venous catheters may be accessed just once a week during clinic visits. We therefore considered that the comparison of the two decontamination regimes in this study to be representative of this latter clinical scenario. A further advantage of this experimental approach was it allowed the longevity of the antimicrobial activity of the cap to be evaluated.

Besides reports of overcoming compliance issues and decreased rates of CLABSI there are several other documented advantages associated with the use of disinfection caps. These include time savings [17], healthcare worker preference [17], a reduction in contamination of blood cultures [9], and cost savings [8, 9, 11].

All these advantages present a persuasive argument to utilise these devices in clinical practice.

## Conclusion

The results of this study support the SHEA/IDSA practice special approach recommendation for preventing CLABSI to ‘use an antiseptic-containing hub/connector cap/port protector to cover connectors (quality of evidence: I)’ [4].

## Abbreviations

BSI: Bloodstream infection
CDC: Centers for Disease Control and Prevention
CFU: Colony forming unit
CHG: Chlorhexidine gluconate
CI: Confidence interval
CLABSI: Central line-associated bloodstream infection
FDA: Food and Drug Administration
IDSA: Infectious Diseases Society of America
IPA: Isopropyl alcohol
NCTC: National Collection of Type Cultures
SHEA: Society for Healthcare Epidemiology of America
TK: Total kill
*v*/*v*: Volume/volume
*w*/*v*: Weight/volume
